# Effects of *Trypanosoma cruzi* on the phenoloxidase and prophenoloxidase activity in the vector *Meccus pallidipennis* (Hemiptera: Reduviidae)

**DOI:** 10.1186/s13071-018-3016-0

**Published:** 2018-07-27

**Authors:** Guadalupe Favila-Ruiz, J. Guillermo Jiménez-Cortés, Alex Córdoba-Aguilar, Paz María Salazar-Schettino, Ana E. Gutiérrez-Cabrera, Armando Pérez-Torres, José Antonio De Fuentes-Vicente, Mauro O. Vences-Blanco, Martha I. Bucio-Torres, A. Laura Flores-Villegas, Margarita Cabrera-Bravo

**Affiliations:** 1 Departamento de Microbiología y Parasitología, Facultad de Medicina, Universidad Nacional Autónoma de México, Ciudad de México, México; 2 Departamento de Ecología Evolutiva, Instituto de Ecología, Universidad Nacional Autónoma de México, Ciudad Universitaria, Apdo. P. 70-275, Circuito Exterior, 04510, Coyoacán, Ciudad de México, México; 3 CONACYT–Centro de Investigación Sobre Enfermedades Infecciosas, Instituto Nacional de Salud Pública, Avenida Universidad 655, Col. Santa María Ahuacatitlán, Cerrada Los Pinos y Caminera, CP 62100 Cuernavaca, Morelos México; 4 Departamento de Biología Celular y Tisular; Facultad de Medicina, UNAM, 04510 Ciudad de México, México; 5 Universidad Pablo Guardado Chávez, Libramiento Norte Poniente #3450, Tuxtla Gutiérrez, Chiapas México

**Keywords:** *Meccus pallidipennis*, *Trypanosoma cruzi*, Phenoloxidase, Prophenoloxidase, Anterior midgut, Hemolymph

## Abstract

**Background:**

Triatomine insects are vectors of *Trypanosoma cruzi*, the causal agent of Chagas disease. The insect-parasite interaction has been studied in relation to the transmission and prevalence of this disease. For most triatomines, however, several crucial aspects of the insect immune response are still unknown. For example, only for *Rhodnius prolixus* and *Triatoma infestans* has the activity of phenoloxidase (PO) and its zymogen prophenoloxidase (proPO) been reported in relation to the hemolymph and anterior midgut (AM). The aim of this study was to gain insight into the immune response to *T. cruzi* infection of an important triatomine in Mexico, *Meccus pallidipennis*.

**Methods:**

Parasites were quantified in the rectal contents of infected *M. pallidipennis* groups. We examined some key factors in disease transmission, including the systemic (hemolymph) and local (gut) immune response.

**Results:**

Parasites were present in the rectal contents at 4 days post-infection (pi) and reached their maximum density on day 7 pi. At 7 and 9 days pi mainly metacyclic trypomastigotes occurred. Compared to the control, the infected insects exhibited diminished PO activity in the hemolymph on days 9, 16 and 20 pi, and in the AM only on day 9. Additionally, infected insects displayed lower proPO activity in the hemolymph on day 1, but greater activity in the AM on day 28.

**Conclusions:**

The parasite strain originating from *M. pallidipennis* rapidly colonized the rectum of nymphs of this triatomine and developed high numbers of metacyclic trypomastigotes. Neither the changes of concentrations of PO and proPO in the hemolymph nor in the AM correlated with the changes in the population of *T. cruzi*.

**Electronic supplementary material:**

The online version of this article (10.1186/s13071-018-3016-0) contains supplementary material, which is available to authorized users.

## Background

Vector-borne parasites and pathogens are major factors in human disease [1]. One example is *Trypanosoma cruzi*, the causal agent of Chagas disease [2]. It is estimated that 7 million people are currently infected with *T. cruzi*, leading to approximately 10,000 deaths annually [3]. The vectors of *T. cruzi* are obligatory hematophagous insects belonging to the subfamily Triatominae [4]. A better understanding of the parasite-triatomine interaction represents one way of attempting to control the propagation of this parasite. The few studies that have focused on the interaction of *T. cruzi* with the triatomine vector are mostly limited to two vector species, *Rhodnius prolixus* and *Triatoma infestans* [5–10]. It is necessary to continue exploring the parasite-vector interaction in relation to other species of the triatomine vector to better understand the post-infection (pi) insect immune response.

*Trypanosoma cruzi* enters the triatomine when it takes a blood meal from an infected mammalian host. This event starts the infection process of the insect host, involving a series of crucial events with important implications for both the parasite and vector. Immediately following ingestion, *T. cruzi* undergoes up to 75% mortality in the anterior midgut (AM) (stomach or crop) of the insect [11]. Despite this reduced initial parasite population; there is an enhanced level of *T. cruzi* in the posterior midgut of the insect by 1–4 weeks pi [11, 12]. Subsequently, the parasite load increases up to 10^6^-fold, reaching a level 25 to 50 times higher in the rectum compared to the posterior midgut [13].

During infection, several morphological changes take place in the intestinal wall that play a key role in the capacity of the parasite to establish itself. In the posterior midgut of the *R. prolixus*, epimastigogenesis involves the division of parasites by binary fusion and their adherence to the perimicrovillar membrane (PMM) of intestinal cells [12, 14]. The latter membrane has diverse adhesion molecules (e.g. a-glycoconjugates and carbohydrates) that act as an adhesion site for the epimastigotes [15].

Afterwards, epimastigotes adhere to the wax cover of the rectal cuticle by hydrophobic interactions, allowing them to transform into metacyclic trypomastigotes (the infectious stage for the mammalian host), which are excreted from the insect in its urine and feces after a blood meal [16–18]. Nymphs of the triatomine *Panstrongylus megistus* are able to transmit *T. cruzi* to vertebrates after 6 to 15 days pi, at which time metacyclic trypomastigotes appear in feces [19].

The high mortality for *T. cruzi* upon entering the triatomine could be due to various local and systemic immune responses of the insect. While the local response should occur in the intestine, the systemic one takes place in the hemolymph [11, 20]. One of the immune responses in insects, the phenoloxidase (PO) cascade, is effective against a wide range of parasites and pathogens [21]. A characteristic of PO is its production of toxic quinones and melanin. By stimulating the cellular immune response, these compounds participate in the phagocytosis of pathogens as well as the sclerotization of the cuticle and healing of wounds, among other processes [22, 23]. The inactive zymogen, prophenoloxidase (proPO), is activated when it recognizes pathogen-associated molecular patterns, which promote the cascade of serine proteases [21]. The final product (melanin) and some intermediates (e.g. reactive oxygen and nitrogen species) encapsulate and kill the pathogenic agent [21, 22]. Both PO and proPO are utilized as robust indicators of the immune capacity of insects [21].

Due to its wide geographical distribution and peridomestic predominance in Mexico, *Meccus pallidipennis* is one of the main triatomine species involved in the transmission of *T. cruzi* [24, 25]. The aim of the present study was to infect this triatomine with *T. cruzi* to gain insights into certain aspects of the insect-parasite interaction: (i) the infective dynamic of the parasite (number of parasites in the rectal contents over time); and (ii) the subsequent immune response of the triatomine measured as PO and proPO activity in both the hemolymph (as a systemic immune response) and the AM (as a local host response).

## Methods

### Nymphs of *Meccus pallidipennis*

In each assay of enzymatic activity, 50 fifth-instar nymphs of *M. pallidipennis* were used per group (control and infected). These insects are native to Oaxtepec, in the State of Morelos, Mexico. Since 1998, they have been reared in the insectarium (at 28 °C, 60% relative humidity and a 12:12 h light/dark photocycle). Insects were fed on strain CD-1 mice (25–30 g) on the Parasite Biology Laboratory in the Department of Microbiology and Parasitology, Faculty of Medicine, Universidad Nacional Autónoma de México.

### Origin of the *Trypanosoma cruzi* isolate

The ITRI/MX/12/MOR strain of *T. cruzi* used presently was given its name according to the terminology of the WHO [26]. It was obtained from an infected male of *M. pallidipennis* (*Triatoma pallidipennis*), originally captured and isolated in 2012 in Cuernavaca, the State of Morelos, Mexico. This strain has been characterized as TCI (R. I. Mendoza Rodríguez, personal communication). The isolate is maintained as a CD-1 model by cyclical passages.

### Insect infection

Forty fifth-instar nymphs of *M. pallidipennis* (starved 15 days after molt) were infected by feeding on CD-1 mice (20–25 g) previously inoculated with a concentration of 20,000 blood trypomastigotes/ml. The mice were used at 20 days pi, which corresponds to the early exponential phase of *T. cruzi* growth. The control group of triatomines was fed on uninfected mice of the same strain and traits. The one-time feeding session (3 insects/mouse) lasted 20 min for both groups (not enabling a total engorgement) and was carried out in dark conditions. Based on the levels of parasitemia in the mice, each infected insect ingested approximately 8000 parasites.

### Confirmation of infection and quantification of parasites

Beginning 12 h pi (day 0.5) and up to 28 days pi, rectal contents were inspected to confirm *T. cruzi* infection [19]. The insects were dissected under a stereoscopic microscope (Stemi 2000 C, Carl Zeiss, Jena, Germany), first legs were removed, and each insect was placed on ice. The abdomen was disinfected with 70% alcohol and cut along the connexivum area to expose the abdominal cavity, removing the Malpighian tubules and all the fat body with entomological calipers. The gut regions were identified [11] and the dissected rectum was transferred to a microtube containing 20 μl physiological saline. The mixture was homogenized with a Vortex mixer (Thomas Scientific 945700, New Jersey, USA) for 1 min. 10 μl were then taken for the quantification of parasites in a Neubauer chamber, employing a dilution of 1:10 [27].

### Extraction and treatment of the hemolymph for phenoloxidase analysis

The hemolymph was extracted on days 0, 1, 4, 7, 9, 16 and 28 from all infected and control insects. After disinfecting the intersection of the third extremity and the thorax, the cuticle was punctured by a 27G needle. Immediately, the abdomen was lightly squeezed for 10 s to induce the flow of the hemolymph through the puncture, modified from [6]. After placing this liquid in a centrifuge microtube (Eppendorf, Hamburg, Germany) previously lined with distilled water, it was diluted 20-fold with PBS (pH 7.2): dibasic sodium phosphate anhydride (Na_2_HPO_4_; 8 × 10^-6^ M), monopotassium phosphate anhydride (KH_2_PO_4_; 1 × 10^-6^ M), potassium chloride (KCl; 3 × 10^-6^ M) and sodium chloride (NaCl; 1 × 10^-4^ M). The tubes were kept on ice during the process and later stored at 4 °C.

### Extraction and treatment of the anterior midgut

At 0.5, 1, 4, 7, 9, 16 and 28 days pi, 40 insects (control and infected) were investigated. Each insect was placed on ice and dissected under a stereoscopic microscope (Carl Zeiss, Stemi 2000 C). The abdomen was disinfected with 70% alcohol and cut along the connexivum. During removal, the intestine ruptured after the cardia. Therefore, the major region of the anterior midgut (AM) (stomach/crop but not the cardia) was deposited in a tube (kept on ice) containing 200 μl PBS (pH 7.2). The sample was macerated with a pestle before being centrifuged at 9168× *g* for 10 min at 4 °C; the supernatant was then diluted 1:10 [10].

### Phenoloxidase and prophenoloxidase activity

PO activity in the hemolymph was determined by spectrophotometry through the catalytic conversion of L-DOPA (3, 4-dihydroxy-L-phenylalanine, which is colorless) to dopachrome (brownish-red), employing the molar extinction coefficient (3.715/M/cm) of the latter [28, 29]. Protein concentrations were quantified in the samples by utilizing the Pierce method with the BCA commercial kit (Thermo Fisher Scientific, Rockford, Illinois, USA) [30]. Hemolymph containing 10 μg of protein was placed in each well of a 96-well microplate (Costar 96; Corning, USA). Then, PBS was added to reach a volume of 100 μl and finally 100 μl L-DOPA substrate (4 mg/ml; Sigma, Sain Louis, Missouri, USA). As a control, 100 μl PBS and 100 μl L-DOPA were used [31]. The mixture was incubated for 20 min at 37 °C in the dark. Readings were taken every 5 min for 1 h at a wavelength of 490 nm to measure PO activity, which was expressed in units of enzymatic activity. The assay was performed in duplicate. These conditions were identical in all determinations of concentrations of PO and proPO.

To determine the activity of proPO, hemolymph containing 10 μg protein was placed in each well of a 96-well microplate. Afterwards, we added PBS to reach a final volume of 65 μl and then 5 μl α-chymotrypsin at a concentration of 1 mg/ml (Sigma, Saint Louis, Missouri, USA) followed by 130 μl L-DOPA. As a control, 5 μl α-chymotrypsin, 65 μl PBS and 130 μl L-DOPA were used [31].

To determine the activity of PO in the AM, 10 μl PBS and 25 μl of the AM sample (previously homogenized) were placed in each well of a 96-well microplate, and then 25 μl L-DOPA were added. As a control, 35 μl PBS and 25 μl L-DOPA were used. The plates were incubated for 3 h at 37 °C in the dark [9, 22].

To establish the activity of proPO in the AM, 45 μl PBS, 25 μl of the AM sample and 5 μl α-chymotrypsin were placed in each well of a 96-well microplate. The mixture was incubated for 1 h at 37 °C, and then 130 μl L-DOPA were added before incubating again for 1 h at 37 °C. As a control, 5 μl α-chymotrypsin, 70 μl PBS and 130 μl L-DOPA were used [31].

### Units of enzymatic activity

A graph (absorbance *versus* time) was constructed and the slope determined. Enzymatic activity was calculated by using the following equation:

$$ \boldsymbol{Enzymatic}\ \boldsymbol{activity}=\frac{m\left(\frac{Abs}{\min}\right)\ast vf(L)\ast F\ }{\varepsilon \left({M}^{-1}{cm}^{-1}\right)\ast b(cm)} $$

In this equation, m is the slope of the graph of absorbance *vs* time (min), vf is the final volume of the reaction expressed in litres, F is the dilution factor, ε is the coefficient of molar extinction for dopachrome (3.715 M^-1^ cm^-1^), and b is the optical path that corresponds to 0.5 cm.

### Statistical analyses

The number of parasites in rectal contents was compared between the control and infected groups by using the Kruskal-Wallis (K-W) test because the data did not show a normal distribution and/or heterogeneity of variance. With the same test we examined possible differences between the two groups in relation to PO and proPO expression in the hemolymph and AM, also due to non-normal distribution of data. The differences between groups for each immune parameter over time were compared with the Mann-Whitney test (M-W). Data are expressed as the mean ± standard error. The analyses were performed and the graphs created with the SPSS program, version 22.

## Results

### Number of parasites in the rectum

**Fig. 1:**
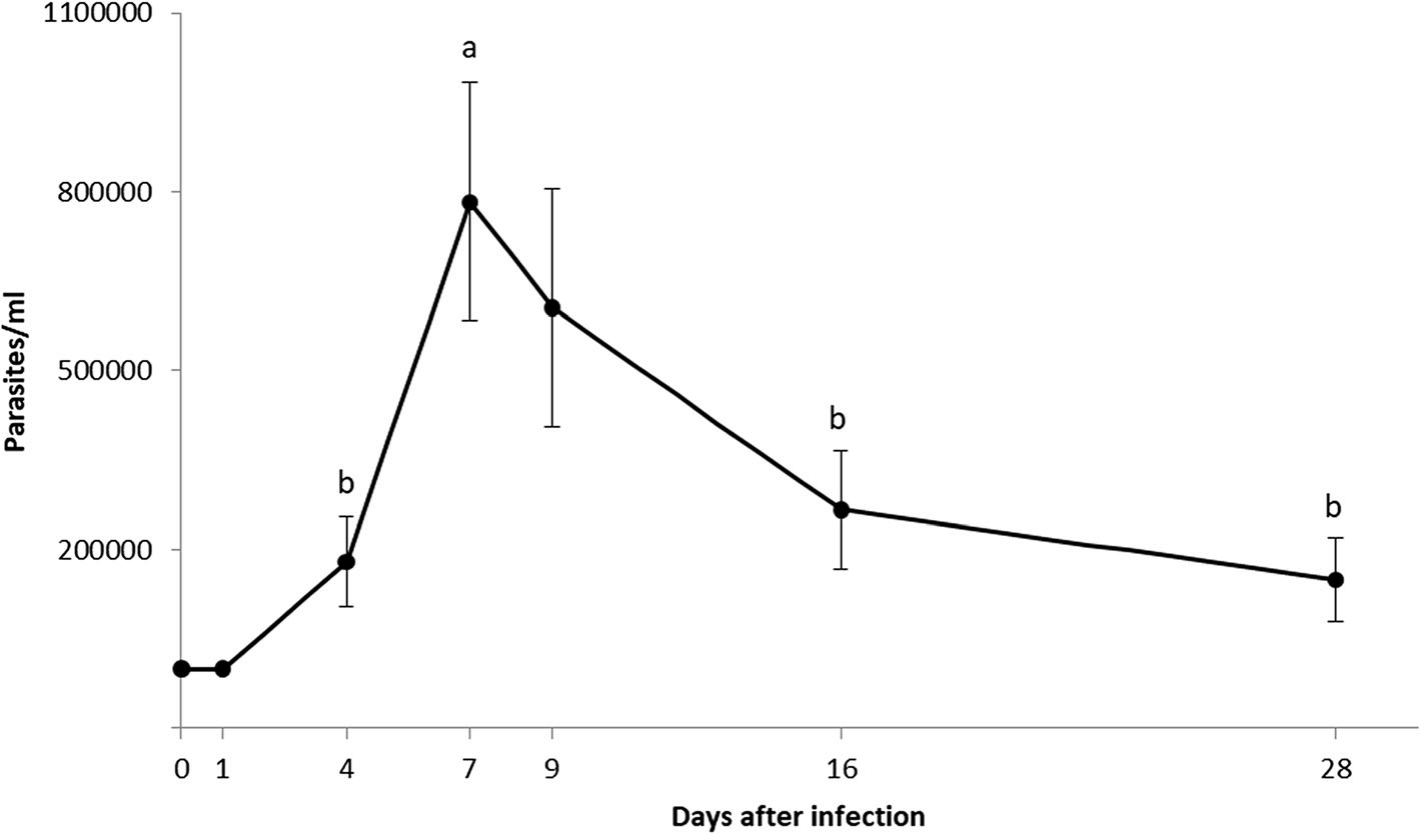
Number of parasites in *M. pallidipennis* nymphs rectal homogenate (*n* = 18 in all cases, except for day 4 where *n* = 15). Different letters indicate a significant difference between these days

Parasites were present in the rectal contents at 4 days pi (Fig. 1). The number of parasites in rectal contents was different between certain end points (K-W *χ*^2^ = 17.304, *P* = 0.002). The number of parasites increased during the first 7 days and then decreased. There was a significant difference between days 4 and 7 (M-W *U* = 66.0, *P* = 0.012), days 7 and 16 (M-W *U* = 64.5, *P* = 0.001), and days 7 and 28 (M-W *U* = 58.5, *P* = 0.001).

### Phenoloxidase and prophenoloxidase activity in the hemolymph

**Fig. 2:**
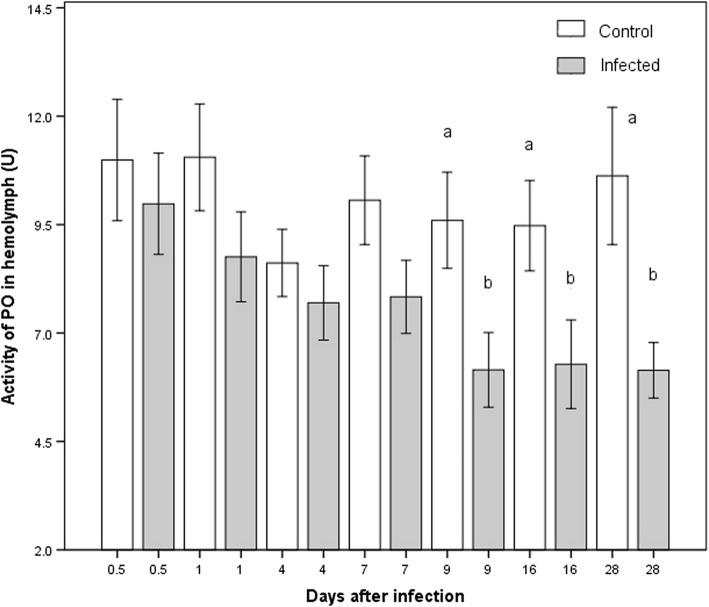
Activity of PO present in the hemolymph of *M. pallidipennis* nymphs. White bars represent data for the control group and gray bars for the infected group. Different letters indicate a significant difference between infected and uninfected nymphs in the respective week

PO activity in the hemolymph gradually decreased over time in infected insects, showing significant differences between some days (K-W, *χ*^2^ = 26.94, *P* = 0.013; Fig. 2). The comparison between the infected group and control revealed significant differences at three points in time (Additional file 1: Table S1): day 9 (M-W *U* = 142.5, *P* = 0.029), day 16 (M-W *U* = 146.5, *P* = 0.043) and day 28 (M-W *U* = 101.0, *P* = 0.036). In all cases, the infected group had lower levels of PO than the control group.

**Fig. 3:**
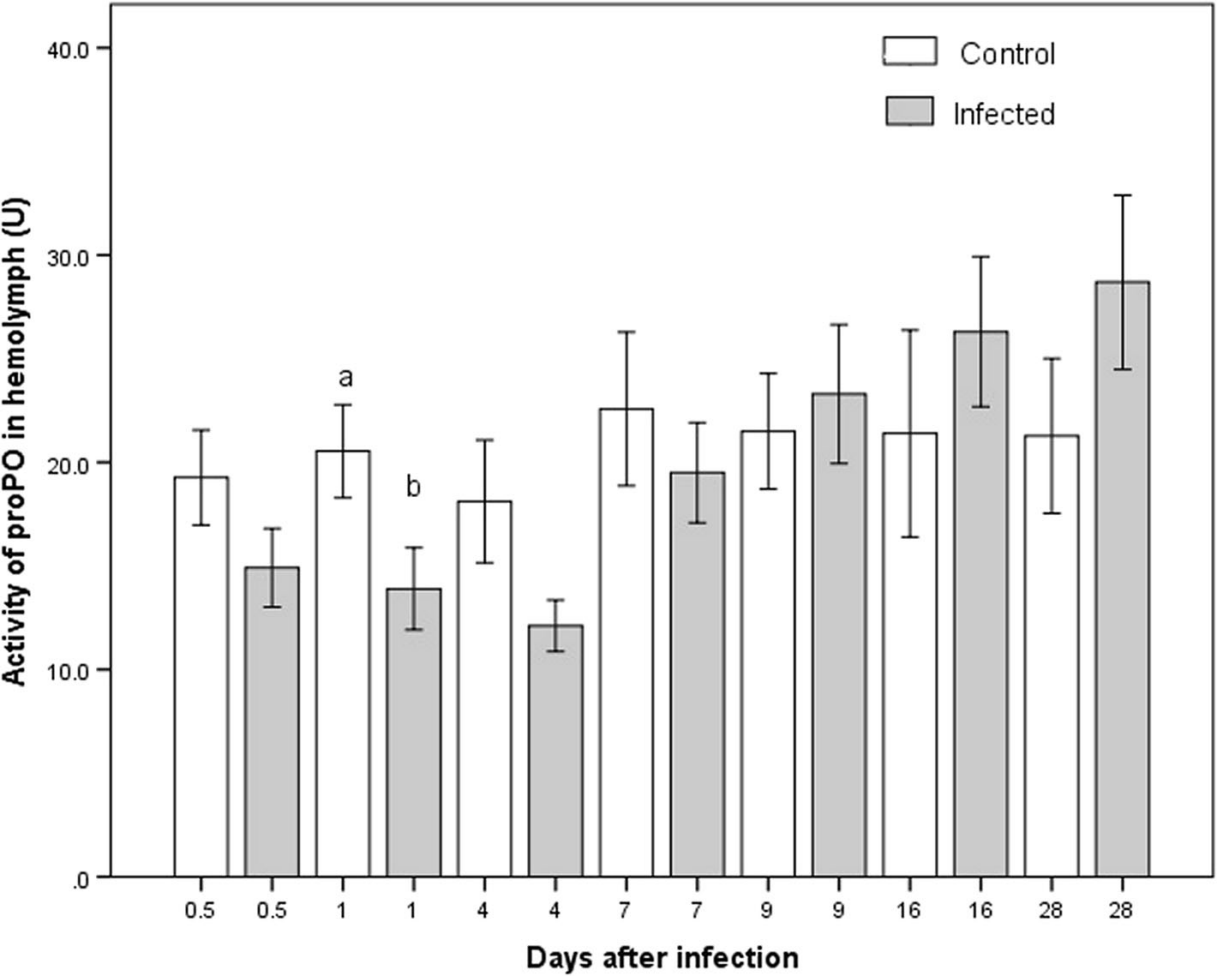
Activity of proPO present in the hemolymph of *M. pallidipennis* nymphs. White bars represent data for the control group and gray bars for the infected group. Different letters indicate a significant difference between infected and uninfected nymphs in the respective week

For infected insects, proPO activity in the hemolymph was different between certain days (K-W *χ*^2^ = 26.61, *P* = 0.014). The lowest activity on day 4 differed statistically significantly from days 7, 9, 16 and 28 pi, which had the highest values (Fig. 3). Day 1 showed the only significant difference between the infected and control groups (Additional file 1: Table S1), with a lower proPO activity in the former (M-W *U* = 165.5, *P* = 0.019).

### Phenoloxidase and prophenoloxidase activity in the anterior midgut

**Fig. 4:**
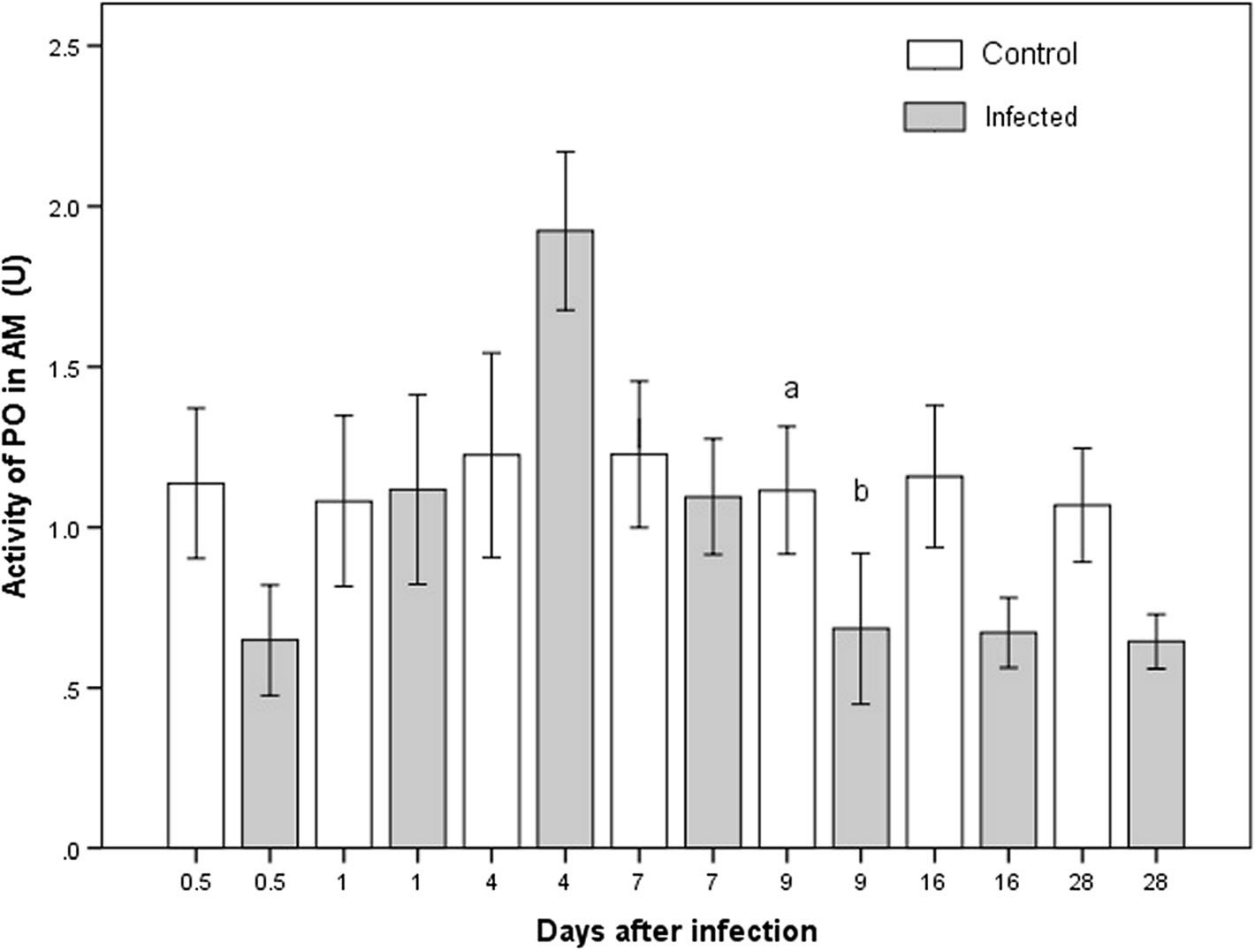
Activity of PO present in the anterior midgut of *M. pallidipennis.* White bars represent data for the control group and gray bars for the infected group. Different letters depict a significant difference between infected and uninfected nymphs in the respective week

In the infected group, PO activity in the AM was significantly different between some time points (K-W *χ*^2^ = 22.99, *P* = 0.042; Fig. 4). The initial values of the infected group were low, rising by day 4 and dropping afterwards. A significant difference existed only on day 9 between the infected and control groups (M-W *U* = 35.0, *P* = 0.033; Additional file 1: Table S1), with a lower PO activity in the former.

**Fig. 5:**
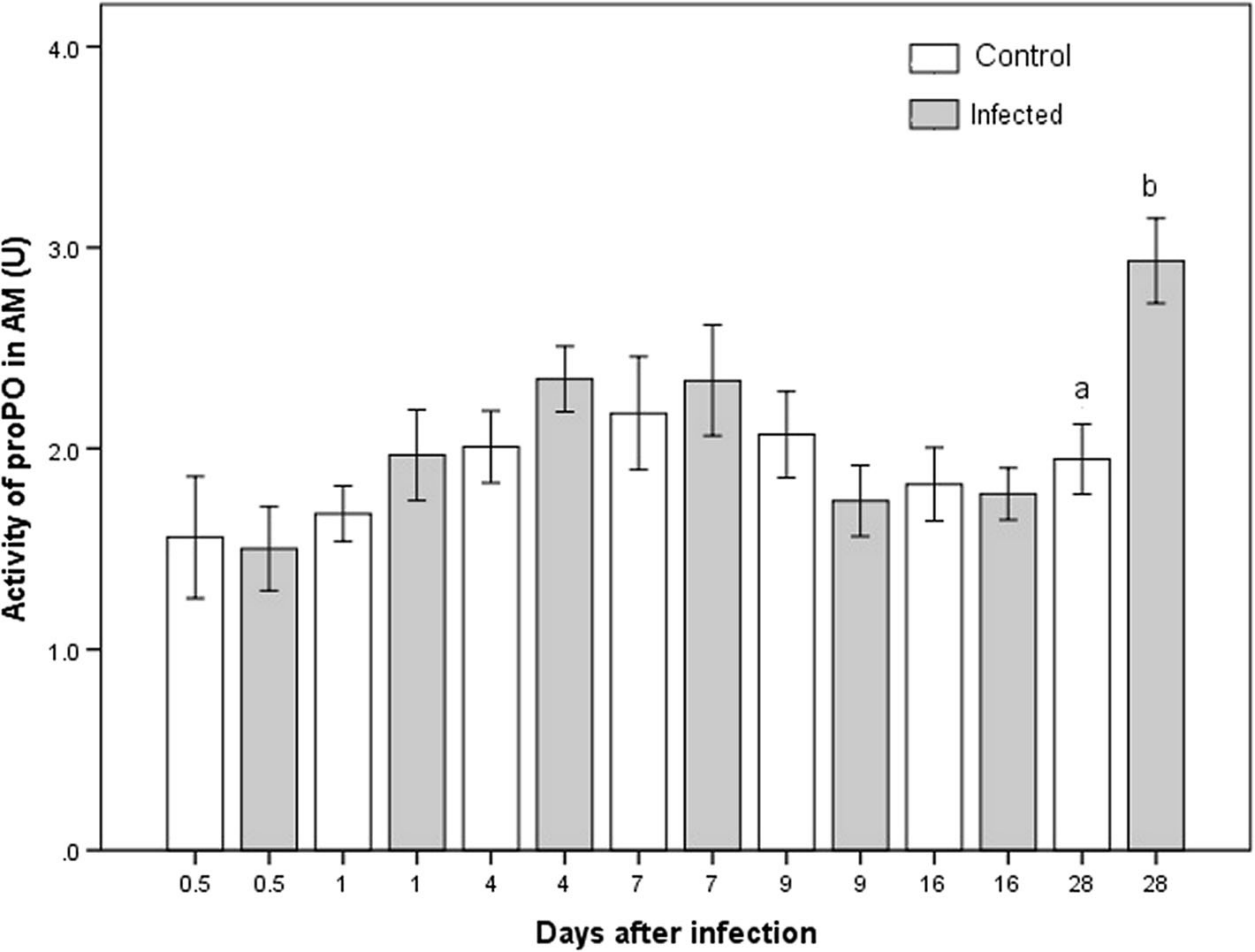
Activity of proPO present in the anterior midgut of *M. pallidipennis*. White bars represent data for the control group and gray bars for the infected group. Different letters indicate a significant difference between infected and uninfected nymphs in the respective week

In the infected group, the level of proPO in the AM varied over time, being elevated in the middle of the experiment, diminishing later and increasing again at the end (K-W *χ*^2^ = 29.28, *P* = 0.006; Fig. 5). No significant difference was detected between the infected and control groups until day 28 (M-W *U* = 41.0, *P* = 0.002; Additional file 1: Table S1), at which time the proPO activity was higher in the former.

## Discussion

After infection of triatomines by *T. cruzi*, metacyclic trypomastigotes appear in the rectal content/feces 2–4 weeks later [32]. In the present model, involving the infection of *M. pallidipennis* with the ITR/MX/12/MOR strain of *T. cruzi*, parasites were present in the rectal contents at 4 days pi and reached their maximum density on day 7 pi. At 7 and 9 days pi mainly metacyclic trypomastigotes occurred. Although this appears to be a short period of time, it coincides with the study using nymphs of *Panstrongylus megistus*, in which *T. cruzi* infection led to the development of the metacyclic form in rectal contents between 6 and 15 days pi [33]. Some nymphs of *Triatoma pseudomaculata* contained metacyclic forms until 30 days pi [34].

The degree of parasitemia detected presently could possibly be related to the immune capacity of *M. pallidipennis*. In fact, PO activity is known to be a response to *T. cruzi* and pathogen infection and triggered at both a local and systemic level [6]. In infected insects, the activity of PO in the hemolymph herein decreased over time. In the AM, it was significantly increased only at 4 days pi, remaining at similar levels afterwards. Also, after infections of *R. prolixus* with the Dm28c strain of *T. cruzi* PO activity in the AM was significantly higher on day 9 pi, compared to the control [10].

In general, our study showed the activity of PO in hemolymph and AM decreases in infected insects, compared with control groups, after the seventh day. It is possible that other components of the immune system in insects, like nitrite production, could be activated 6–12 h pi, as seen in *R. prolixus* infected with *T. cruzi* [35].

However there are other causes that could explain decrease of PO in *M. pallidipennis* in the following days pi; the production and maintenance of PO is known to be largely dependent on the condition of the insect [21]. First, PO activation is highly dependent of insects feeding conditions; the amino acid phenylalanine is the precursor of melanization cascade that is activated by PO [22, 23]. Secondly, during the activation of PO, a great quantity of reactive oxygen (ROS) and reactive nitrogen species (RNS) are generated, which may be toxic to the host. Such an effect has been demonstrated in other insect taxa [22, 36]. Thirdly, an effect of a detachment of resident hemocytes should be considered by a determination of the concentration of hemocytes in the hemolymph and the PO activity of the hemocytes and the hemolymph [37].

The activity of proPO in the hemolymph was significantly different between the infected and control groups only on the first day pi, being greater in the latter. This could have been caused by an large immune response during the first few days of infection, implying that a large part of proPO would be utilized as PO. At some point, the concentration of proPO in tissues would have to stabilize and increase, given its multiple functions in the organism. In the AM, proPO activity tended to be more erratic, perhaps as a consequence of fluctuations in parasite density. The parasite population might have been diminishing on certain days as a result of the immune response, while rising on others due to the rapid binary division of epimastigotes. Whereas the former situation should generate an enhanced activity of proPO, the latter would activate the PO cascade and lead to a decrease in proPO [15].

## Conclusions

Parasite counts in infected insects showed a rapid development of *T. cruzi* in *M. pallidipennis*, with the greatest abundance on day 7 pi. The immune response of the triatomine, on the other hand, was in part represented by PO activity, which was detected at the local (AM) and systemic (hemolymph) level. Hence, significant differences between infected and uninfected insects may illustrate the effect of the parasite on the vector. Whereas the systemic response decreased during the entire 28 days of the study, the local response increased during the first four days. Another immune response parameter was proPO, the zymogen of PO. ProPO was more erratic than PO in both the hemolymph and AM, perhaps because of its multiple functions in insect physiology.

## Additional file

Additional file 1: **Table S1.** Activity of PO and proPO in *Meccus pallidipennis*, according to hemolymph or anterior midgut and group. (DOCX 21 kb)

## Abbreviations

AM: anterior midgut
K-W: Kruskal-Wallis test
M-W: Mann-Whitney test
pi: post-infection
PO: phenoloxidase
proPO: prophenoloxidase

## References

[1] Molyneux DH (2006). Control of human parasitic diseases: context and overview. Adv Parasitol..

[2] Dias JCP. Chagas disease (American trypanosomiasis). In: Marcondes CB, editor. Arthropod Borne Diseases. Berlin: Springer International Publishing; 2017. p. 245–75.

[3] World Health Organization (2017). Chagas disease (American trypanosomiasis).

[4] World Health Organization (2014). A Global Brief on Vector-Borne Disease.

[5] Gregório EA, Ratcliffe NA. The proPO system and in vitro interactions of *Trypanosoma rangeli* with *Rhodnius prolixus* and *Triatoma infestans* haemolymph. Parasite Immunol. 1991;13:551–64. 10.1111/j.1365-3024.1991.tb00551.x 1956701

[6] Mello CB, Garcia ES, Ratcliffe NA, Azambuja P (1995). *Typanosoma cruzi* and *Trypanosoma rangeli*: interplay with hemolymph components of *Rhodnius prolixus*. J Invertebr Pathol..

[7] Gomes SAO, Feder D, Garcia ES, Azambuja P (2003). Suppression of the proPO system in *Rhodnius prolixus* orally infected with *Trypanosoma rangeli*. J Insect Physiol..

[8] Vallejo GA, Guhl F, Schaub GA (2009). Triatominae-*Trypanosoma cruzi*: vector-parasite interactions. Acta Trop..

[9] Vieira CS, Mattos DP, Waniek PJ, Santangelo JM, Figueiredo MB, Gumiel M (2015). *Rhodnius prolixus* interaction with *Trypanosoma rangeli*: modulation of the immune system and microbiota population. Parasit Vectors..

[10] Castro DP, Moraes CS, Gonzalez MS, Ratcliffe NA, Azambuja P, Garcia ES (2012). *Trypanosoma cruzi* immune response modulation decreases microbiota in *Rhodnius prolixus* gut and is crucial for parasite survival and development. PLoS One..

[11] Schaub GA, Meiser CK, Balczun C. Interactions of *Trypanosoma cruzi* and triatomines. In: Mehlhorn H, editor. Progress in Parasitology. Berlin: Springer; 2011. p. 155–78.

[12] Ferreira RC, Kessler RL, Lorenzo MG, Paim RM, de l FL, Probst CM (2016). Colonization of *Rhodnius prolixus* gut by *Trypanosoma cruzi* involves an extensive parasite killing. Parasitology..

[13] Kollien AH, Schaub GA (2000). The development of *Trypanosoma cruzi* in Triatominae. Parasitol Today..

[14] Gonzalez MS, Nogueira NFS, Mello CB, De Souza W, Schaub GA, Azambuja P, Garcia ES (1999). Influence of brain and azadirachtin on *Trypanosoma cruzi* development in the vector, *Rhodnius prolixus*. Exp Parasitol..

[15] Azambuja P, Garcia ES (2005). *Trypanosoma rangeli* interactions within the vector *Rhodnius prolixus*: a mini review. Mem Inst Oswaldo Cruz..

[16] Garcia ES, Ratcliffe NA, Whitten MM, Gonzalez MS, Azambuja P (2007). Exploring the role of insect host factors in the dynamics of *Trypanosoma cruzi*-*Rhodnius prolixus* interactions. J Insect Physiol..

[17] Garcia ES, Genta FA, Azambuja P, Schaub GA (2010). Interactions between intestinal compounds of triatomines and *Trypanosoma cruzi*. Trends Parasitol..

[18] Guarneri AA, Lorenzo MG (2017). Triatomine physiology in the context of trypanosome infection. J Insect Physiol..

[19] Zeledón R. Infection of the insect host by *Trypanosoma cruzi*. In: Carcavallo RU, Galíndez-Giron I, Jurberg J, Lent H, editors. Atlas of Chagas Disease Vectors in the Americas. Rio de Janeiro: Editora Fiocruz; 1997. p. 271–98.

[20] Flores-Villegas AL, Salazar-Schettino PM, Córdoba-Aguilar A, Gutiérrez-Cabrera AE, Rojas-Wastavino GE, Bucio-Torres MI, Cabrera-Bravo M (2015). Immune defence mechanisms of triatomines against bacteria, viruses, fungi and parasites. Bull Entomol Res..

[21] González-Santoyo I, Córdoba-Aguilar A (2012). Phenoloxidase: a key component of the insect immune system. Entomol Exp Appl..

[22] Söderhäll K, Cerenius L (1998). Role of the proPO-activating system in invertebrate immunity. Curr Opin Immunol..

[23] Genta FA, Souza RS, Garcia ES, Azambuja P (2010). Phenol oxidases from *Rhodnius prolixus*: temporal and tissue expression pattern and regulation by ecdysone. J Insect Physiol..

[24] Ibarra-Cerdeña CN, Sánchez-Cordero V, Towsend-Peterson A, Ramsey JM (2009). Ecology of North American Triatominae. Acta Trop..

[25] Salazar-Schettino PM, Rojas-Wastavino GE, Cabrera-Bravo M, Bucio-Torres MI, Martínez-Ibarra JA, Monroy-Escobar MC (2010). Revisión de 13 especies de la familia Triatominae (Hemiptera: Reduviidae) vectores de la enfermedad de Chagas, en México. J Selva Andina Res Society..

[26] WHO Expert Committee on the Control of Chagas Disease (2000: Brasilia, Brazil) & World Health Organization. Control of Chagas disease: second report of the WHO expert committee. Geneva: World Health Organization. 2002. http://www.who.int/iris/handle/10665/42443.

[27] Reis DD, Monteiro WM, Bossolani GDP, Teston APM, Gomes ML, Marques de Araújo S (2012). Biological behaviour in mice of *Trypanosoma cruzi* isolates from Amazonas and Paraná, Brazil. Exp Parasitol..

[28] Laughton AM, Siva-Jothy MT (2010). A standaraised protocol for measuring PO and proPO in the honey bee, *Apis mellifera*. Apidologie..

[29] Daquinag AC, Nakamura S, Takao T, Shimonishi Y (1995). Primary structure of a potent endogenous DOPA-containing inhibitor of phenol oxidase from *Musca domestica*. Proc Natl Acad Sci USA..

[30] Flores-Villegas AL, Cabrera-Bravo M, Toriello C, Bucio-Torres MI, Salazar-Schettino PM, Córdoba-Aguilar A (2016). Survival and immune response of the Chagas vector *Meccus pallidipennis* (Hemiptera: Reduviidae) against two entomopathogenic fungi, *Metarhizium anisopliae* and *Isaria fumosorosea*. Parasit Vectors..

[31] Jiménez-Cortés JG, Córdoba-Aguilar A. Condition dependence and trade-offs of sexual *versus *non-sexual traits in an insect. J Ethol. 2013;31:275–84.

[32] Schaub GA (1989). *Trypanosoma cruzi*: quantitative studies of development of two strains in small intestine and rectum of the vector *Triatoma infestans*. Exp Parasitol..

[33] Piesman J (1985). *Trypanosoma cruzi*: kinetics of metacyclogenesis in adult and nymphal *Panstrongylus megistus*. Exp Parasitol..

[34] Perlowagora-Szumlewicz A, Moreira CJ. In vivo differentiation of *Trypanosoma cruzi* - 1. Experimental evidence of the influence of vector species on metacyclogenesis. Mem Inst Oswaldo Cruz. 1994;89:603–18. 10.1590/s0074-02761994000400018 8524063

[35] Whitten M, Sun F, Tew I, Schaub G, Soukou C, Nappi A, Ratcliffe N. Differential modulation of *Rhodnius prolixus* nitric oxide activities following challenge with *Trypanosoma rangeli*, *T. cruzi* and bacterial cell wall components. Insect Biochem Mol Biol. 2007;37:440–52. 10.1016/j.ibmb.2007.02.001 17456439

[36] Lu A, Zhang Q, Zhang J, Yang B, Wu K, Xie W, Ling E (2014). Insect proPO: the view beyond immunity. Front Physiol..

[37] Whitten MMA, Mello CB, Gomes SAO, Nigam Y, Azambuja P, Garcia ES, Ratcliffe NA (2001). Role of superoxide and reactive nitrogen intermediates in *Rhodnius prolixus* (Reduviidae)/*Trypanosoma rangeli* interactions. Exp Parasitol..

