# Supplementary material for: Effects of Trypanosoma cruzi on the phenoloxidase and prophenoloxidase activity in the vector Meccus pallidipennis (Hemiptera: Reduviidae)
Source: Parasit Vectors. 2018 Jul 27;11:434. doi: 10.1186/s13071-018-3016-0 (PMC6062883; doi:10.1186/s13071-018-3016-0)
Supplement: Supplementary file 1 — Table S1. Activity of PO and proPO in Meccus pallidipennis, according to hemolymph or anterior midgut and group. (DOCX 21 kb) [file 13071_2018_3016_MOESM1_ESM.docx]

**Additional file 1: Table S1** Activity of PO and proPO in *Meccus pallidipennis*, according to hemolymph or anterior midgut and group

| **Day** | **PO in**  **H, C** | **PO in**  **H, I** | **proPO in**  **H, C** | **proPo in**  **H, I** | **PO in**  **AM, C** | **PO in**  **AM, I** | **proPO in**  **AM, C** | **proPO in**  **AM, I** |
| --- | --- | --- | --- | --- | --- | --- | --- | --- |
| **0.5** | 10.99+1.40 | 9.98+1.17 | 19.27+2.29 | 14.92+1.90 | 1.14+0.234 | 0.64+0.17 | 1.56+0.30 | 1.5+0.20 |
| **1** | 11.05+1.23 | 8.76+1.04 | **20.54+2.24** | **13.90+1.96** | 1.08+0.27 | 1.12+0.29 | 1.67+0.14 | 1.97+0.23 |
| **4** | 8.62+0.77 | 7.69+0.86 | 17.69+3.02 | 12.12+1.24 | 1.22+0.32 | 1.92+0.25 | 2.01+0.18 | 2.34+0.16 |
| **7** | 10.06+1.02 | 7.83+0.84 | 22.58+3.71 | 19.51+2.41 | 1.23+0.22 | 1.09+0.18 | 2.17+0.28 | 2.34+0.27 |
| **9** | **9.6+ 1.11** | **6.15+ 086** | 21.5+2.79 | 23.3+3.36 | **1.11+0.19** | **0.68+0.23** | 2.07+0.21 | 1.74+0.17 |
| **16** | **9.47+1.04** | **6.28+1.02** | 21.4+4.99 | 26.31+3.62 | 1.16+0.22 | 0.67+0.11 | 1.82+0.183 | 1.77+0.13 |
| **28** | **10.62+1.58** | **6.14+0.64** | 21.28+3.73 | 28.69+4.19 | 1.07+0.17 | 0.64+0.09 | **1.95+0.17** | **2.93+0.21** |

Values (mean ± standard error)

*Abbreviations:* H Hemolymph, AM Anterior midgut, C Control, I Infected

The bold type indicated a significant difference between the control and infected groups
